# Pattern of mRNA expression of β-defensins in basal cell carcinoma

**DOI:** 10.1186/1471-2407-6-163

**Published:** 2006-06-23

**Authors:** T Gambichler, M Skrygan, J Huyn, FG Bechara, M Sand, P Altmeyer, A Kreuter

**Affiliations:** 1 Department of Dermatology and Allergology, Ruhr-University Bochum Gudrunstr. 56, D-44791 Bochum, Germany

## Abstract

**Background:**

Although the human β-defensins hBDs today seem to have diverse functional activities in innate antimicrobial immunity, a few reports also indicated an altered expression of these antimicrobial peptides (AMPs) in tissues of cancers such as oral squamous cell carcinoma. The present work was aimed on the study of hBD gene expression in basal cell carcinoma (BCC) which is the most common cancer in humans.

**Methods:**

Twenty-two non-ulcerated BCCs (12 nodular type, 10 superficial type) have been analysed for the presence of hBD (1–3) mRNA by quantitative real-time RT-PCR. As controls, non-lesional skin specimens of BCC patients as well as samples of healthy subjects were assessed by RT-PCR.

**Results:**

hBD-1 levels in healthy controls and non-lesional skin of BCC patients were significantly (P < 0.05) higher than the levels observed in tumour tissue. Moreover, BCCs showed significantly (P < 0.05) increased mRNA expression of hBD-2 as compared to controls. There was no significant (P > 0.05) difference between lesional mRNA levels for hBD-3 and those levels observed in controls. The mRNA expression of hBDs (1–3) found in nodular and superficial BCCs did not significantly (P > 0.05) differ.

**Conclusion:**

The gene expression patterns of hBD-1 and hBD-2 are for the first time shown to be significantly altered in non-ulcerated BCCs as compared to intra-individual and inter-individual controls, respectively. The present findings may indicate that beside the antimicrobial activity of AMPs, hBDs may also play a role in the pathogenesis of BCC. However, functional and immunohistological studies investigating hBDs in patients with BCC are needed to confirm our data.

## Background

Basal cell carcinoma (BCC) is the most common cancer in humans. It is classified, together with squamous cell carcinoma, as non-melanoma skin cancer. The incidence of BCC is increasing worldwide by up to 10% a year. Although mortality is low as BCC rarely metastasises, this malignancy causes considerable morbidity and places a huge burden on healthcare services worldwide. Three main types of BCCs are generally distinguished with regard to the histopathological growth pattern: nodular, superficial, and morphoeic. BCCs are believed to derive from the epidermis, specifically the basal cell layer and the outer root sheath of the hair follicle. The development of BCC is clearly associated with mutated p53 tumour-suppressor gene and constitutive activation of sonic hedgehog signalling molecules that regulate cell proliferation and cell fate determination. Risk factors for the development of BCC include advanced age, fair skin colour, chronic ultraviolet exposure, and immunosuppression [1,2].

BCCs are 10 times more common in persons who have undergone a solid organ transplant and herpes-virus like DNA sequences have been found in these tumours. The possibility that certain types of human papilloma virus, either alone or in conjunction with ultraviolet radiation, may play a role in the pathogenesis of particular cancers is suggested by several lines of evidence [3]. The human β-defensins (hBDs) are antimicrobial peptides (AMPs) mainly produced in skin by keratinocytes, neutrophils, and mast cells. In addition to their strong antimicrobial activity, hBDs may act as chemoattractant of neutrophils, monocytes, and T lymphocytes [4]. Although hBDs today seem to have diverse functional activities in innate antimicrobial immunity, a few reports also indicated an altered expression of these AMPs in tissues of different cancers [5-9]. In this pilot study we aimed to investigate the expression of hBDs in patients with BCC and healthy controls.

## Methods

### Subjects

In this prospective pilot study, 28 patients (16 men and 12 women; median age: 79.4; range: 64–96 years), who were suspected of having non-ulcerated BCC on the basis of clinical and dermoscopic features, underwent complete tumour excision in our dermatologic surgery. Three millimetre punch biopsies were harvested from the centre of the tumour (lesional) as well as an adjacent healthy skin site approximately 1 cm in distance to the tumour border (non-lesional controls). In addition to the BCC patient group, we included 27 subjects (12 men and 15 women; median age: 62.3 years; range: 27–83 years) who underwent cosmetic surgery (healthy controls). Punch biopsies were taken from the centre of excised skin tissue that was otherwise rejected. We have not controlled age and gender for the healthy subjects, since these parameters do not significantly influence AMP expression. However, we sought to obtain skin specimens from facial as well as body skin in controls to exclude any bias that may arise from anatomic variations of AMP expression [4]. The main portion of each BCC specimen was fixed in formalin, routinely processed, and embedded in paraffin. Sections were stained with haematoxylin and eosin. All punch biopsy specimens were processed for real-time RT-PCR analysis. The study was conducted in the light of the declaration of Helsinki. All patients who participated in the investigation signed an informed consent.

### Real-time RT-PCR

**Figure 1:**
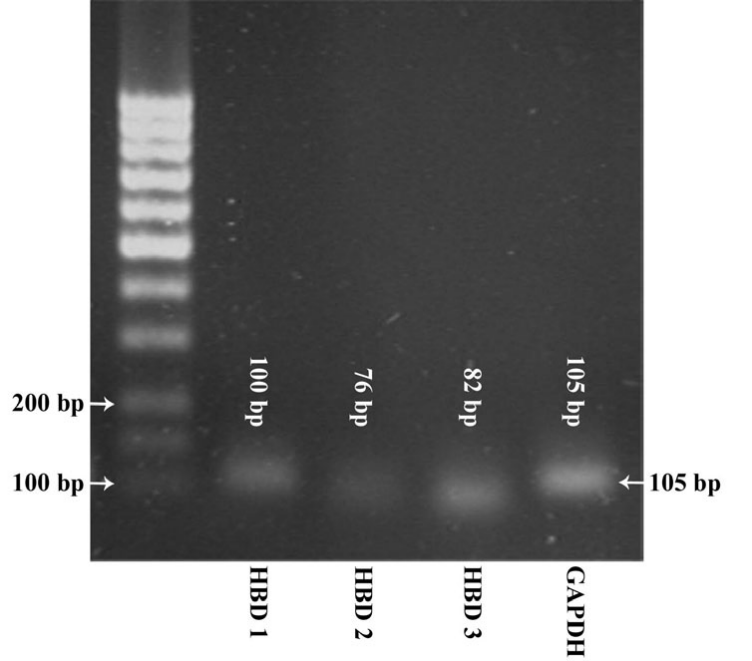
PCR products of hBDs and housekeeping gene (GAPDH) on 2% agarose gels.

Quantitative analysis of real-time RT-PCR was performed as previously suggested [10]: total cellular RNA was isolated from skin tissue samples using RNeasy^® ^Lipid Tissue Kit (QIAGEN, Chatsworth, CA) following the manufacturer's protocol. Prior to cDNA synthesis RNA was digested with RNase-free DNase I (Roche Diagnostics, North America). cDNA was synthesized by reverse transcription from DNase I treated RNA using MultiScribe™ reverse transcriptase enzyme and random hexamers primers (TagMan^® ^Reverse transcription reagents, Applied Biosystems, Forster City, CA). Real-time PCR was performed using a Taqman SYBR Green PCR Master Mix and GeneAmp^® ^Sequence Detection System (Applied Biosystems). PCR Primers for AMPs and the Housekeeping Gene (GAPDH) were designed using the computer program Primer Express (PE Biosystems) and produced by the custom oligonucleotide synthesis service TIB Molbiol (Germany). The primers for hBDs (1–3) and GAPDH are given in Table 1. PCR amplifications were performed in a total volume of 25 μl, containing 5 μl cDNA sample, 5 μM of each primer and 12.5 μl SYBR Green PCR Master Mix. PCR was started with 2 min at 50°C and an initial 10 min denaturing temperature of 95°C, followed by a total of 40 cycles of 15 sec of denaturing and 1 min of annealing and elongation at 60°C. The reaction products were separated by 2% agarose gel electrophoresis (Fig. 1). Relative expression levels were calculated by the relative standard curve method as outlined in the manufacturer's technical bulletin. The comparative Δ-ΔC_t _method was used as previously suggested by Livak and Schmittgen [11]. A standard curve was generated using the fluorescent data from 10-fold serial dilutions of total RNA of the highest expression sample. This was then used to calculate the relative amounts of target mRNA in test samples. Quantities of all targets in the test samples were normalized to the corresponding GAPDH RNA transcript in the skin samples. In order to make the quantities of mRNA levels more illustrative, the ΔC_t _values (logarithms) were re-transformed.

### Statistics

Data analysis was performed using the statistical package MedCalc Software (Mariakerke, Belgium). Non-normal distribution of data was confirmed by the D'Agostino-Pearson test. Hence, data were expressed in medians and lowest and highest values (range). The results were analysed using paired or independent non-parametric tests including the Wilcoxon-rank test and the Mann-Whitney test. We constrained experiment-wise error rates due to multiple comparisons to the standard alpha (P) level of < 0.05 by the Bonferroni method.

## Results

**Figure 2:**
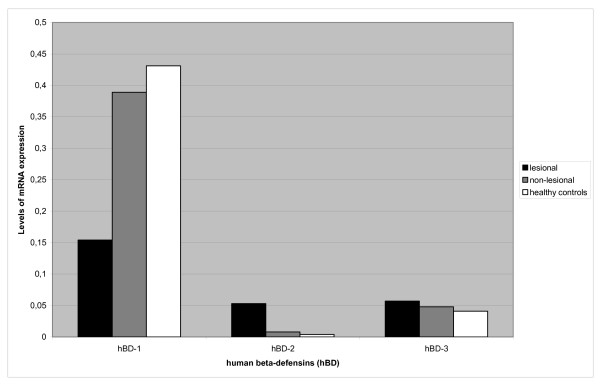
**Diagram showing levels of median mRNA expression of hBDs in lesional and non-lesional skin of 22 patients with non-ulcerated basal cell carcinoma.** hBD mRNA expression in 27 healthy controls is also shown. mRNA expression levels given on the y-axis correspond to the re-transformed logarithms of the ΔCt values.

Fifteen of the BCCs investigated were localised on the face and 13 on the trunk and lower extremities. Histological examination revealed 18 nodular BCCs and 10 superficial BCCs. Six of the nodular BCCs on the face showed ulceration on histology and were therefore excluded from further evaluation. In the control group, we harvested 11 biopsies from the face and 16 on the trunk and lower extremities. The medians and range of mRNA expression of AMPs in BCC patients (n = 22) and healthy controls (n = 27) are shown in Fig. 2 and Table 1, respectively. The mRNA levels of hBDs (1–3) observed in healthy skin of controls did not significantly (P > 0.05) differ from non-lesional hBD levels of BCCs patients. However, hBD-1 levels in healthy controls and non-lesional skin were significantly (P < 0.05) higher than the hBD-1 levels observed in BCCs. Moreover, BCCs showed significantly (P < 0.05) increased mRNA expression of hBD-2 as compared to healthy controls and non-lesional skin. There was no significant (P > 0.05) difference between lesional mRNA levels for hBD-3 and those levels observed in healthy controls and non-lesional skin. The mRNA expression of hBDs (1–3) found in nodular and superficial BCCs did not significantly (P > 0.05) differ (hBD-1: 0.140 vs 0.290; hBD-2: 0.061 vs 0.0451; hBD-3: 0.050 vs 0.028).

## Discussion

Current information on AMP structure, expression, and biologic activity has established a firm foundation for further study of the impact that these peptides might have in human disease. For example, human α-defensin (hAD) expression has previously been linked to different types of tumours and cell lines. hAD-1 has been detected in lung tumours and in the submandibular glands of patients with oral carcinomas. By RT-PCR, mass spectrometry and flow cytometric analysis, hADs (1–3) have been shown to be expressed by cell lines deriving from renal cell carcinomas and the expression of a specific hAD precursor peptide has been shown to be upregulated in human leukemic cells. It has been suggested that hADs could belong to tumour substrates that modulate malignant cell growth and enhance immune escape in tumours [12-14]. Moreover, several reports recently indicated an altered expression of hBDs in tissues of different cancers including oral squamous cell carcinoma, lung cancer, renal and prostatic carcinoma, and vulval cancer. However, to our best knowledge, there are no reports exploring the significance of AMPs in the pathogenesis of skin cancer such as BCC [5-9].

We have shown that mRNA expression of hBDs insignificantly differ between non-lesional skin of BCC patients and healthy controls indicating that there exists no constitutional difference in hBD gene expression. Nevertheless, we observed significantly altered expression of hBD-1 and hBD-2 in BCCs as compared to controls. The alterations observed in BCCs were however independent from tumour subtypes. The hBD gene expression patterns observed in BCCs do not appear to be substantially different from those seen in inflammatory conditions such as atopic dermatitis and psoriasis [4,15,16]. HBD-1 belongs to the constitutive β-defensins which has been observed in normal epidermis. However, hBD-1 is not increased in response to inflammation, as is hBD-2, and displays antimicrobial activity against Gram-positive and Gram-negative bacteria, as well as viral agents [4,15]. Interestingly, we found significantly decreased mRNA levels of hBD-1 in BCC possibly indicating that expression of this constitutive AMP is markedly downregulated in tumour tissue. Notably, in previous gene expression profiling studies of renal epithelial neoplasms, hBD-1 was found to be significantly downregulated in conventional clear cell carcinoma [7]. In contrast to its weak antimicrobial activity, hBD-1 has a strong cytotoxic potential toward mammalian cells, leading to speculation that hBD-1 had functions other than the antimicrobial activity so characteristic of other defensins [7,17]. Furthermore, the expression of hBD-1 early in development suggests that it has physiologic functions rather than only host microbial defense. Considering the existing literature together with the data presented in this paper, one my speculate that hBD-1 may contribute to host antitumour immunity or otherwise function as a tumour suppressor gene. If hBD-1 expression is lost at some point during the malignant transformation, the host may be less likely to recognize the tumour as "foreign", and the tumour may be more likely to survive [7]. Nevertheless, our data do not address these functional questions.

The virtual absence of hBD-2 in controls observed in the present study, gives support to previous reports that this AMP is not normally produced but are upregulated under pathological conditions such as inflammation (e.g., bacterial infection, atopic eczema, psoriasis) and malignancies (e.g., oral squamous carcinoma). We have demonstrated that hBD-2 levels are significantly upregulated in BCC. Although we sought to include into the study only BCC without evidence of ulceration, we cannot fully exclude that impairment of skin barrier functions associated with an increased invasion of microbes, contributed to the upregulation of hBD-2 in tumour tissue investigated. Whereas impairment of skin barrier functions are common in BCC, it has been demonstrated that germ colonization of BCC lesions is usually indifferent from normal skin [18,19]. Yoshimoto et al. [5] performed a study on oral squamous cell carcinoma and found that the expression of hBD-2 was observed not only in inflamed lesions with bacterial infection but also in the non-inflamed carcinomas themselves. The authors concluded that hBD-2 might play a role in squamous cell carcinoma, which is different from the native defensive role of AMP. Indeed, one may speculate that hBD-2 could lead to the death of normal keratinocytes adjacent to the tumour, which might, in turn, indirectly assist in the multiplication of malignant cells [5,9]. Unlike hBD-1 and hBD-2, lesional hBD-3 levels insignificantly differed from controls as observed in the present study. Shnitsar et al. [8] recently investigated hBD-3 gene expression in A431 cell line and human vulval tumours. Interestingly, they found that in human vulval epithelium, the increase in hBD-3 mRNA expression was predominantly associated with malignant phenotype.

## Conclusion

The gene expression patterns of hBD-1 and hBD-2 are for the first time shown to be significantly altered in non-ulcerated BCCs as compared to intra-individual and inter-individual controls, respectively. The heterogeneity of hBD expression in BCC indicates that beside the antimicrobial activity of AMPs, hBDs have other unique functions that may also play a role in the pathogenesis of BCC. The altered expression of hBDs by transformed keratinocytes may impair host immune recognition and control of cellular proliferation and/or differentiation [20,21]. The altered expression of hBDs in epithelial cancers such as BCC, on the other hand, could also be a result of impaired proliferation and/or differentiation of keratinocytes [5,8,9]. However, this was not specifically investigated in our study. Indeed, limitations of the present trial do not only include the absence of functional, but also immunohistological studies. The latter could confirm the presence and localisation of differentially expressed genes on the protein level as well. Hence, our results have to be substantiated by functional and immunohistological investigations on hBDs in patients with BCC.

## Abbreviations

BCC: basal cell carcinoma; AMP: antimicrobial peptide; hBD: human β-defensin; hAD: human α-defensin.

## Competing interests

The author(s) declare that they have no competing interests.

## Authors' contributions

TG drafted the manuscript, participated in the design of the study, and performed the statistical analysis and interpretation of data. MS carried out the molecular genetic studies and participated in the sequence alignment. JH, FGB and MS obtained the biological samples. AK conceived of the study and participated in its design and coordination. All authors read and approved the final manuscript.

## Pre-publication history

The pre-publication history for this paper can be accessed here:



## Figures and Tables

**Table 1:** Primers used in the PCR study and medians (range) of mRNA expression of hBDs in patients with basal cell carcinoma and healthy controls.

**Primer**	**Sequence**		
*hBD-1*	F 5'-AGATGGCCTCAGGTGGTAACTTT-3'; R 5'-GGGCAGGCAGAATAGAGACATT-3'		
*hBD-2*	F 5'-TGATGCCTCTTCCAGGTGTTT-3'; R 5'- GGATGACATATGGCTCCACTCTT-3'		
*hBD-3*	F 5'-TCCATTATCTTCTGTTTGCTTTGC-3'; R 5'-TTCTGTAATGTGTTTATGATTCCTCCAT-3'		
*GAPDH*	F 5'-CCTCAACTACATGGTTTACATGTTCC-3'; R 5'-ATGGGATTTCCATTGATGACAAG-3'		
			

**Medians and range of mRNA expression**			

	**lesional**	**non-lesional**	**healthy controls**

*hBD-1*	0.154 (0.002 – 0.634)	0.389 (0.111 – 0.941)	0.431 (0.161 – 0.981)
*hBD-2*	0.053 (0.000 – 0.974)	0.008 (0.001 – 0.162)	0.004 (0.000 – 0.178)
*hBD-3*	0.057 (0.002 – 0.915)	0.048 (0.006 – 0.263)	0.041 (0.004 – 0.541)

hBD, human β-defensin; GAPDH, Glyseraldehyde-3-phosphate dehydrogenase
